# Recent Updates on Phytoconstituent Alpha-Glucosidase Inhibitors: An Approach towards the Treatment of Type Two Diabetes

**DOI:** 10.3390/plants11202722

**Published:** 2022-10-14

**Authors:** Hamdy Kashtoh, Kwang-Hyun Baek

**Affiliations:** Department of Biotechnology, Yeungnam University, Gyeongsan 38541, Korea

**Keywords:** α-glucosidase, postprandial hyperglycemia, natural compounds, type 2 diabetes

## Abstract

Diabetes is a common metabolic disorder marked by unusually high plasma glucose levels, which can lead to serious consequences such as retinopathy, diabetic neuropathy and cardiovascular disease. One of the most efficient ways to reduce postprandial hyperglycemia (PPHG) in diabetes mellitus, especially insulin-independent diabetes mellitus, is to lower the amount of glucose that is absorbed by inhibiting carbohydrate hydrolyzing enzymes in the digestive system, such as α-glucosidase and α-amylase. α-Glucosidase is a crucial enzyme that catalyzes the final stage of carbohydrate digestion. As a result, α-glucosidase inhibitors can slow D-glucose release from complex carbohydrates and delay glucose absorption, resulting in lower postprandial plasma glucose levels and control of PPHG. Many attempts have been made in recent years to uncover efficient α-glucosidase inhibitors from natural sources to build a physiologic functional diet or lead compound for diabetes treatment. Many phytoconstituent α-glucosidase inhibitors have been identified from plants, including alkaloids, flavonoids, anthocyanins, terpenoids, phenolic compounds, glycosides and others. The current review focuses on the most recent updates on different traditional/medicinal plant extracts and isolated compounds’ biological activity that can help in the development of potent therapeutic medications with greater efficacy and safety for the treatment of type 2 diabetes or to avoid PPHG. For this purpose, we provide a summary of the latest scientific literature findings on plant extracts as well as plant-derived bioactive compounds as potential α-glucosidase inhibitors with hypoglycemic effects. Moreover, the review elucidates structural insights of the key drug target, α-glucosidase enzymes, and its interaction with different inhibitors.

## 1. Introduction

**Scheme 1:**
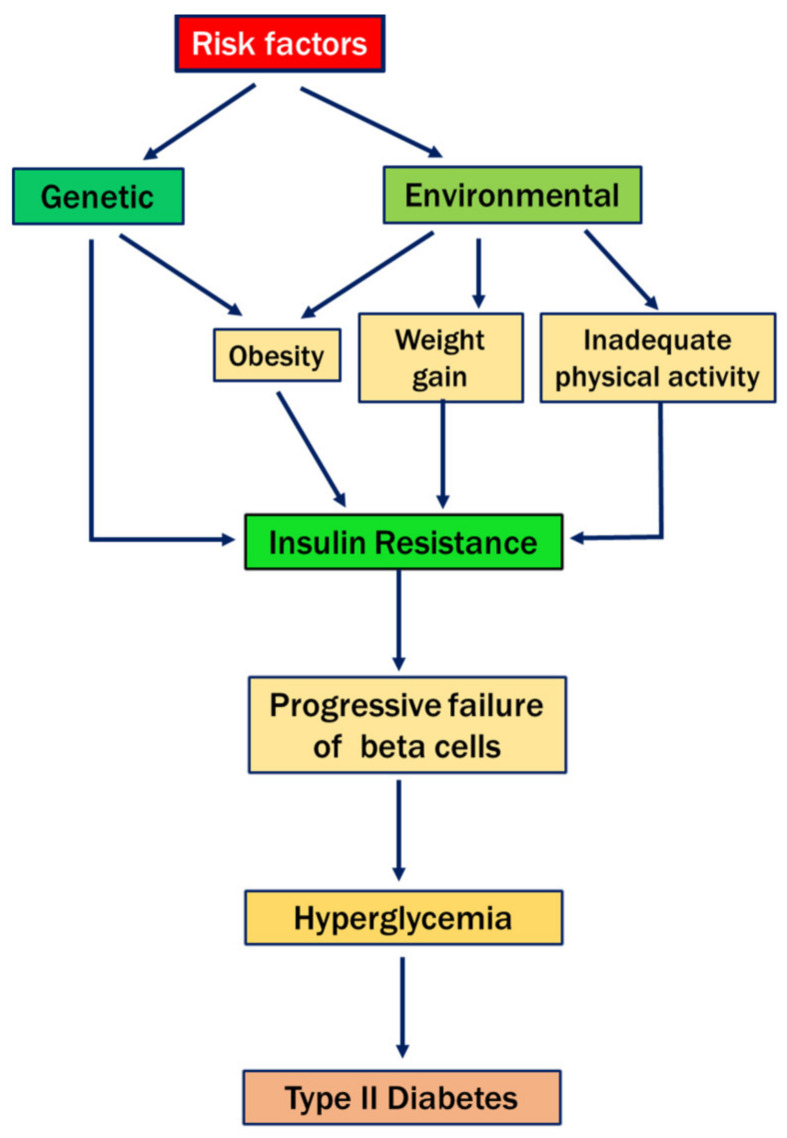
Flow chart for the pathophysiology of type 2 diabetes.

Diabetes mellitus is a metabolic condition defined by chronically high blood sugar levels [1]. The International Diabetes Federation Diabetes Atlas estimates that it affected 537 million people globally in 2021, and that number is expected to rise to 643 million by 2030 [2]. Diabetes mellitus was the ninth major cause of mortality in a worldwide study conducted by the World Health Organization (WHO) (2019), and it is projected to be the seventh leading cause of death by 2030. According to the International Diabetes Federation (IDF), 6.05 million individuals in Korea suffer from diabetes mellitus as of 2020 [3]. The insulin hormone is generated by pancreatic β-cells and plays a key role in regulating blood glucose levels. It is required for several cellular activities such as glucose absorption and transport, glycogen synthesis, protein synthesis and fatty acid synthesis. Inadequate insulin production or insulin resistance hinders proper glucose homeostasis, resulting in hyperglycemia [4]. Chronic hyperglycemia can have major long-term consequences such as cardiovascular disease nerve damage and renal failure [5]. Depending on the mechanism of its manifestation, diabetes mellitus can be categorized into three types; type 1 diabetes mellitus (T1DM), type 2 diabetes mellitus (T2DM) and gestational diabetes. T1DM affects roughly 5–10% of all diabetes patients and is characterized by the death of pancreatic insulin-producing β-cells destroyed by the immune system, resulting in an extreme shortage of insulin, hyperglycemia, inflammation, oxidative damages and other metabolic problems [6,7]. T2DM affects over 90% of diabetes people worldwide and is expected to reach 592 million by 2035 [8]. T2DM is characterized by insulin resistance resulting from insulin receptor insensitivity, persistent hyperglycemia, dyslipidemia and low-level inflammation (Scheme 1) [8,9]. Gestational diabetes occurs only during pregnancy in women and results in unfavorable clinical conditions in both the mother and her kids [10]. Hyperglycemia is the most serious criterion of all forms of diabetes, and it can lead to a variety of complications such as cardiovascular disease, neuropathy, renal failure, lipid metabolism issues and many others. Therefore, controlling blood glucose levels in diabetes individuals is very critical [11,12]. Reduced postprandial hyperglycemia is one treatment method for treating diabetes in its early stages. This is accomplished by suppressing the carbohydrate-hydrolyzing enzymes, α-glucosidase and α-amylase in the digestive system, which prevents glucose absorption. As a result, inhibitors of these enzymes slow the absorption of glucose, hence dampening the postprandial plasma glucose spike [13,14].

Since the 1990s, anti-diabetic medicines with α-glucosidase inhibitory capabilities, such as acarbose, miglitol and voglibose, have been commercially accessible for treating postprandial hyperglycemia. Since their molecular structure is comparable to that of disaccharides or oligosaccharides, those antidiabetic drugs can bind to the carbohydrate-binding site of α-glucosidase. The complexes that result from such binding have a higher affinity than carbohydrate–glucosidase complexes, which consequently leads to a delay in carbohydrate digestion and absorption and thus reduces the PPHG. Nonetheless, the repeated ingestion of them causes flatulence, severe stomach discomfort, allergic responses, etc. [15,16,17]. Despite the commercial availability of efficient AGIs, researchers are continuously working developing novel bioactive AGIs with strong inhibitory potential and fewer adverse effects. Several bioactive compounds have been reported to alleviate various pathophysiological conditions [18,19,20,21,22,23,24,25,26]. Additionally, numerous attempts have been made to synthesize non-cytotoxic compounds with α-glucosidase inhibition activity [27,28,29]. In recent decades, there has been a surge of growing interest in using natural products as medicinal agents, particularly in the prevention and management of T2DM. Medicinal herbs and traditional remedies have been employed throughout history to treat a wide range of medical conditions, including diabetes. This review gives an overview of the most recent plant-derived extracts as well as bioactive compounds that inhibit α-glucosidase, and it emphasizes the most promising therapeutic candidates for T2DM management via α-glucosidase inhibition. The most recent updates include, from various natural sources, different plant extracts, their hypoglycemic effect on animal models, phenolic compounds, flavonoids, tannins, anthocyanins and polysaccharides. The review was carried out based on published work between 2019 and 2022 by using scientific search engines such as Scopus, PubMed, Science Direct and SciFinder. The inclusion criteria were medical plants with a folklore history exhibiting α-glucosidase activities.

## 2. Alpha-Glucosidases Structure and Mechanism of Action

**Figure 1:**
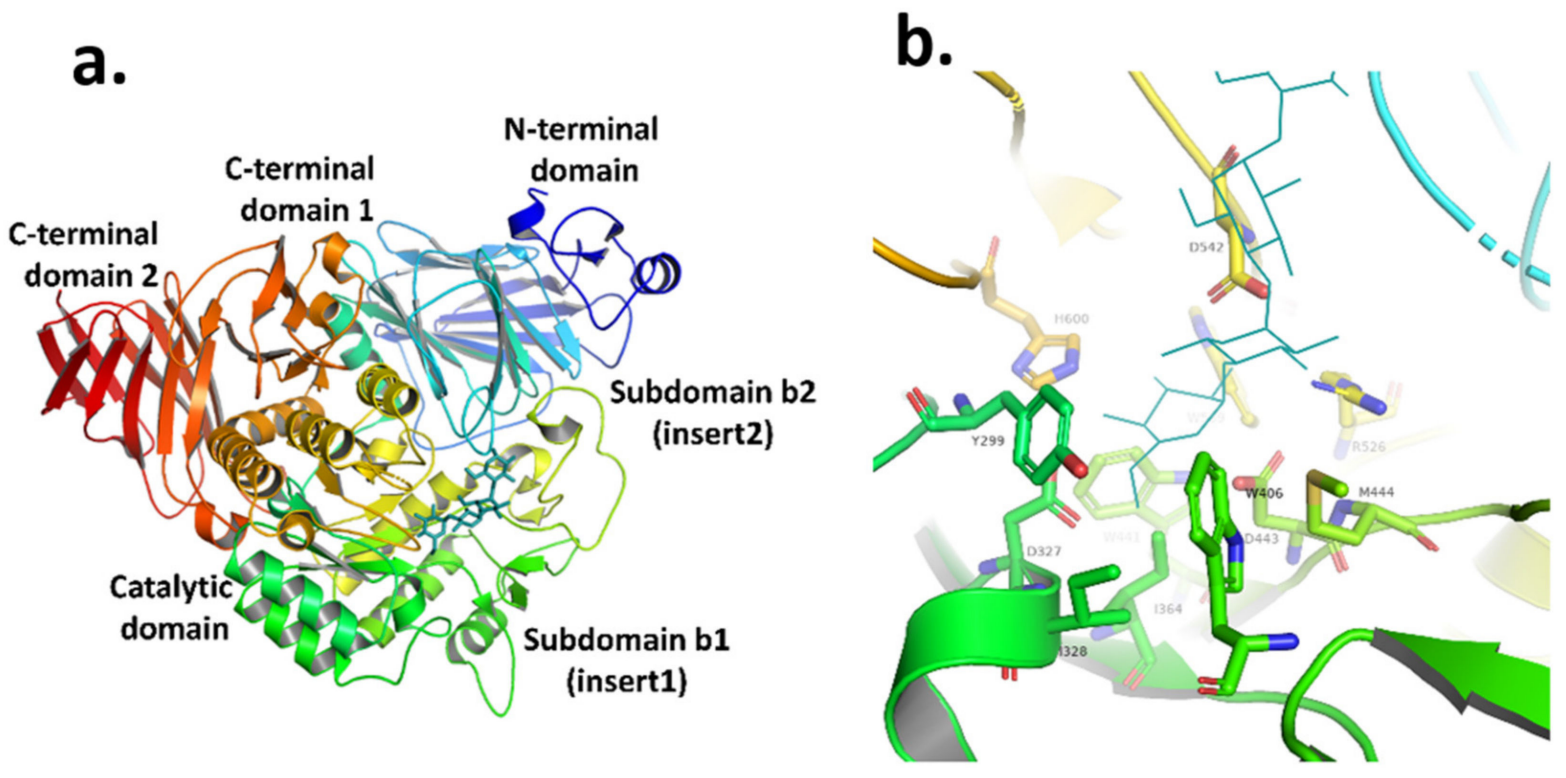
(**a**) Ribbon diagram of the structure of Human Nt MGAM/acarbose complex as a representative for GH31 α-glucosidase. Different domains are colored as follows: N-terminal domain, blue; catalytic domain, green; subdomain b1, pale green; subdomain b2, lemon; C-terminal domain 1, red; and C-terminal domain 2, orange. (**b**) Human Nt MGAM/acarbose complex active site; sticks represent residues situated within a 4-A° radius of a valienamine unit. The acarbose is colored cyan and is shown as sticks and wire for a and b, respectively. (**a**,**b**) were adopted from the structure, with PDB entry code: 2QMJ [39], and were generated using PyMol [40].

Complex carbohydrates are broken down into monosaccharides in the gastrointestinal system by several breakdown processes and are absorbed in the small intestine. The digestive process starts with the production of amylases (EC 3.2.1.1), which catalyze the breakdown of starch into shorter polysaccharides and are mostly generated by the salivary and pancreatic glands [30]. When partly hydrolyzed starch enters the small intestine, it is further processed by amylases of the pancreas, which target the α-1 and four linkages of carbohydrate-releasing dextrins [31]. α-Glucosidases at the brush border of enterocytes mediate the last stage in glucose metabolism. The enzymes have duplicated glycoside hydrolase domains (GH31) that hydrolyze α-glucosidic disaccharide and oligosaccharide bonds [32,33] (Figure 1a). These glycosidases play important roles in a variety of biological activities, including carbohydrate digestion, lysosomal glycoconjugate catabolism and post-translational glycoprotein changes. The oligosaccharides resulting from α-amylase digestion are finally hydrolyzed to monosaccharides by α-glucosidases; maltase glucoamylase [MGAM (EC 3.2.1.20) and (EC 3.2.1.3)] and sucrose isomaltase [SI (EC 3.2.1.48) and (EC 3.2.1.10)]. MGAM (EC 3.2.1.20) are the most active of the four α-glucosidases, releasing glucose from non-reducing ends of oligosaccharides [34,35,36,37,38].

**Figure 2:**
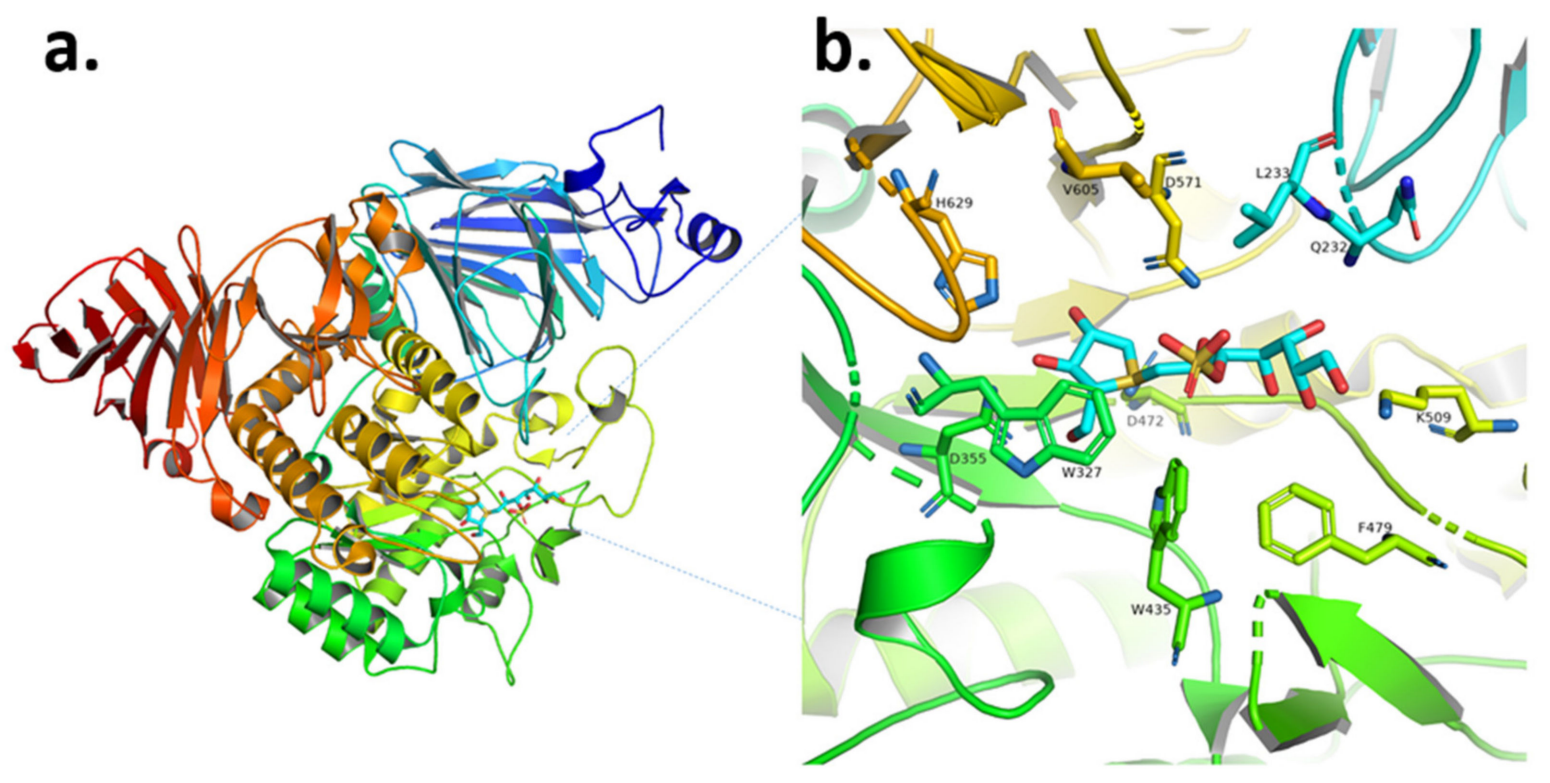
(**a**) Ribbon diagram of Human Nt SI crystal structure in complex with kotalanol. (**b**) Human Nt SI important active site residues (catalysis/substrate binding). The kotalanol is colored cyan and is shown as sticks. (**a**,**b**) were adopted from the structure, with PDB entry code: 3LPP [43], and were generated using PyMol [40].

**Figure 3:**
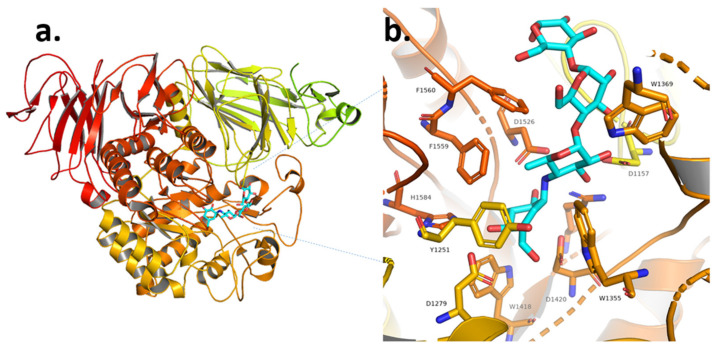
(**a**) Ribbon diagram of the structure of human ctMGAM/acarbose complex. (**b**) Human ctMGAM important active site residues (catalysis/substrate binding). The acarbose is colored cyan and is shown as sticks. (**a**,**b**) were adopted from the structure, with PDB entry code: 3TOP [44], and were generated using PyMol [40].

The catalytic domains of MGAM and SI are duplicated, with an N-terminal membrane-adjacent domain (ntMGAM and ntSI) and a C-terminal luminal domain (ctMGAM and ctSI) (Figure 1a, Figure 2a and Figure 3a). An O-glycosylated stalk produced from the N-terminal domain attaches the domains to the brush border membrane of the small intestine [41]. The N- and C-terminal domains of MGAM and SI have more sequence similarity (~60%) when compared to the N- and C-terminus domains of the same enzyme in other species (~40 percent sequence identity). This is due to the MGAM and genes evolving from a previously duplicated ancestor gene through duplication and divergence. The N- and C-terminals of MGAM and SI are members of the glycoside hydrolases (GH) 31 family. The nonreducing ends of α (1–4)-glycosidic bonds are hydrolyzed by the four domains, although they have different inclinations for malto-oligosaccharides of variant lengths [35,36,37,38]. MGAM favors α-1,4-oligosaccharides and can effectively hydrolyze lengths up to glucohexaose. α-1, 6-glycosidic linkages are hydrolyzed by MGAM at just a 2% rate compared to α-1,4-glycosidic bondage, and there is a little sum of α-1,2- and α-1,3-hydrolyzing activity. On the other hand, SI represents almost 80% of the total intestinal maltase activity (α-1,4 glycosidic linkages) and nearly all sucrase activity (α-1,2-glycosidic linkages) in the small intestine. SI may also hydrolyze isomaltose’s α-1,6-glycosidic bonds, and there is modest α-1,3-hydrolyzing activity [41,42]. The hydrolyzed glucose is then transported by glucose transporter (GLUT)-2 and sodium/glucose cotransporter-1 (SGLT1) from intestinal mucosa into the blood circulation, causing postprandial hyperglycemia (PPHG) [38].

Since the inhibition of α-glucosidase enzymes results in a glucose production delay, which contributes to its therapeutic role in T2DM, the relationship between α-glucosidases’ catalytic characteristics, particularly substrate selectivity, and their structures have been the subject of much research in the past two decades. Except for CtSI, the three-dimensional structures of these subunits are now available [39,43,44]. The α-glucosidases’ structures are protein complexes containing inhibitors such as acarbose and kotalanol. (Figure 1, Figure 2 and Figure 3). Each α-glucosidase structure consists of four main domains; an N-terminal domain, a catalytic domain of the (the (β/α)_8_-barrel and two C-terminal domains. Inserts 1 and 2 of the catalytic domain are located right after β-strands 3 and 4, respectively (Figure 1a). The general architectures of these subunits’ structures are almost similar, except for insert 1. CtMGAM insertion 1 differs from the others because it includes an additional helical segment of 21 amino acid residues [44] (Figure 3a). In the catalytic domain, the active site pocket (Subsite-1) is formed by β-barrel loops, and the residues involved with subsite-1 formation are highly conserved among α-glucosidases’ subunits. At subsite-1, twelve residues reside within 4-A° of an acarbose valienamine unit and may contribute to enzyme/inhibitor interactions (Y299, D327, I328, I364, W406, W441, D443, M444, R526, W539, D542 and H600) (Figure 1b). D443 and D542 each supply a catalytic nucleophile and a generic acid/base. The hydroxy groups of the valienamine establish a hydrogen bond with the side chains of D327, R526 and H600 (Figure 1b). In NtMGAM, the aromatic residue of Y299 of the catalytic domain is oddly different. Both MGAM subunits feature Tyrosine residue (Y299 in NtMGAM and Y1251 in CtMGAM) (Figure 1b and Figure 3b), and NtSI has W327 (Figure 2b). This Tryptophan residue is thought to be key in giving the α-(1→6)-specificity of NtSI [43] (Figure 2b). Mutational studies have shown that substituting Tryptophan residues for the Y299 of NtMGAM and Y1251 of CtMGAM enhances the enzyme catalytic activity for isomaltose hydrolysis [44]. The binding of α-glucosidase with isomaltose (α-(1→6) specific) was clarified using the crystal structure of α-glucosidase from *Ruminococcus obeum* [45]. The W169 bulky side chain appeared to impede its mobility by being opposed to the flexible α-(1→6)-glucosidic linkage with three bonds. A site-directed mutagenesis investigation demonstrated the relevance of W169 to α-(1→6)-specificity, in which the replacement of W169 with Y significantly lowered the hydrolysis activity toward isomaltose and turned the α-(1→6) specific α-glucosidase into an α-(1→4)-specific enzyme [45]. These structural insights can help us to understand α-glucosidase interactions with different AGI to produce AGI with fewer side effects.

## 3. Plant Extracts as α-Glucosidase Inhibitor Sources

Many herbal medications have been advocated for diabetes treatment in addition to the already available therapeutic alternatives. Traditional plant remedies are utilized all over the world to treat a variety of diabetes symptoms. The fact that plant preparations have fewer adverse reactions than current conventional medications [46,47,48,49], along with their lower cost, is encouraging both the general population and national health care organizations to examine natural medical items as alternatives to synthetic drugs [50]. As a result, research into such compounds derived from traditional medicinal herbs has become increasingly significant [51].

*Cucurbitaceae* family member *Momordica charantia* L. has been exploited as a traditional medicine for managing diabetes mellitus and other metabolic syndromes [52]. *M. charantia* is rich in phytoconstituents such as flavonoids, alkaloids, polysaccharides, poly peptides, glycosides phenolic and fatty acids that enhance its pharmacologic efficacy [53,54]. *M. charantia* methanolic extract shows potent α-glucosidase inhibition activity and significantly improves fasting blood glucose levels and insulin in diabetic rats. The acarbose shows higher α-glucosidase inhibition (79.91 ± 0.77%) in vitro than *M. charantia* methanolic extract (72.30 ± 0.30%) [52].

*Artemisia absinthium* belongs to the *Asteraceae* family, which is considered to be the most common traditional Moroccan medicine used for diabetes [55]. The hypoglycemic effect of *A. absinthium* L. aqueous and ethyl acetate extracts have been studied in diabetic rats [56]. *A. absinthium* ethyl acetate extracts show higher α-glucosidase inhibition activity in vitro than the aqueous extract (IC_50_ for ethyl acetate extract 0.155 ± 0.0009 mg/mL, aqueous extract 0.170 ± 0.0002 mg/mL as compared to acarbose 0.148 ± 0.002 mg/mL). However, in vivo, only the aqueous extract of *A. absinthium* leaves show significant hypoglycemic activity, whereas the ethyl acetate extract shows no α-glucosidase activity. Such activity could be due to the high content of polyphenols in the *A. absinthium* extract.

Several extracts (20) from edible spices such as mace, nutmeg, coriander, star anise and fenugreek were investigated for their anti-diabetic potential as α-glucosidase inhibitors [57]. Among them, the ethyl acetate extract of star anise has the most potent anti-α-glucosidase activity in vitro (IC_50_ 4.76 ± 0.71 to 201.34 ± 20.07 μg/mL of control acarbose). The mechanism of inhibition was further investigated, and the kinetic analysis revealed the competitive and reversible binding of star anise ethyl acetate extract to α-glucosidase. The study showed that star anise ethyl acetate extract injection in hyperglycemic rabbits decreases blood glucose levels significantly and in a time-dependent manner.

*Amomum villosum* plant fruit from the *Zingiberaceae* family is a Korean traditional medicine used in the treatment of different digestive diseases. The fruit water extract used by healthy individuals shows a positive effect on postprandial glycemia and insulin secretion during clinical assessment [58]. *A. villosum* water extract was investigated for its α-glucosidase activity at different concentrations of 1, 3 and 5 mg/mL, which proportionally increased the inhibition against rat α-glucosidase with IC50 of 31.99 ± 6.79%, 48.85 ± 4.75% and 62.58 ± 6.69%, respectively. Although *A. villosum* water extract has lower inhibition on α-glucosidase than the reference acarbose, it showed a considerable drop in blood glucose levels in the sucrose loading test when administered to the rats compared to the control group [59].

*Merremia tridentata* (L.) is a traditional medicinal plant used for the treatment of diabetes and several other disorders in Vietnam. The antidiabetic effect of stem-ethanol extract (SE) as well as flavonoid-rich fractions (FF) of the stem of *M. tridentata* were investigated in diabetic mice [60]. The study revealed that the daily administration of 100 mg/kg SE and 50, 75 mg/kg FF to diabetic mice for twenty days has a higher hypoglycemic effect than the reference drugs, metformin (10 mg/kg) and glibenclamide (5 mg/kg), without affecting the body weight of tested mice. Moreover, SE and FF showed decent α-glucosidase inhibition activity when compared with acarbose (IC_50_ (mg/mL) 0.44 ± 0.11, 0.24 ± 0.08 and 0.29 ± 0.06, respectively) (Table 1).

Several medicinal plant extracts have been recently reported to exhibit potent α-glucosidase inhibitory activity and hypoglycemic effects in animal models. For one of the most famous and commercial green teas in China (Lu’an guapian green tea (LGGT)), its methanol extract shows α-glucosidase inhibition activity, and when supplemented with the diet, it improves insulin sensitivity and glucose tolerance in mice [61]. For another edible spice/medicinal herb from China, *Amomum tsao-ko*, its methanol extract shows hypoglycemic activity in a dose-dependent manner while treating STZ-induced diabetic mice as well as in vitro [62]. After six weeks of treatment, the extract significantly decreases the fasting blood glucose in diabetic mice. The study identifies bioactive constituents from methanol extracts such as phenols, flavonoids, oligosaccharides, coumarins and others that could be responsible for α-glucosidase inhibition/hypoglycemic activity. Recently, edible and hydroponically grown *Lactuca sativa* soil have been reported to substantially reduce blood glucose levels in diabetic rats besides in vitro α-glucosidase inhibition activity [63]. The crude extract and two isolated compounds Coniferol (1) and dillapiole (2) (from chloroform phyto-fractions) of *Allium consanguineum* were investigated for their hypoglycemic effects [64]. The in vivo studies revealed that two compounds, coniferol and dillapiole, substantially lower blood glucose levels in albino mice. The ethanolic leaves extract of *Amischotolype mollissima* has shown α-glucosidase enzymatic activity in addition to the antihyperglycemic effect that was observed in the swiss albino mice oral glucose tolerance test in a dose-dependent manner [65]. The methanolic flower extract of *Descurainia sophia* showed in vitro α-glucosidase activity with mixed (competitive/non-competitive) inhibition [66]. Moreover, consuming the flower extract reduced blood glucose levels in the male rats when compared to the control group. The authors propose that the hypoglycemic effect of the *D. sophia* flower extract is due to flavonoid and phenolic phytochemical contents in the extract (Table 1).

Other traditional plant extracts have been recently reported for their α-glucosidase potency, and further in vivo studies are required to verify their hypoglycemic biological effect. These studies have examined the potential role of herbal plants against α-glucosidase activity (Table 2). Among the most recent plant extract studies in the literature that are included in this review, *Cerasus humilis*, *Gymnanthemum amygdalinum*, and *Paliurus spina-christi* Mill have the highest α-glucosidase inhibition activities compared to the positive control acarbose. *Cerasus humilis* (Sok. leaf-tea) has been identified as a good source of α-glucosidase inhibitors [84]. *C. humilis* methanol extract with a high flavonoid/phenolic content has a substantially higher α-glucosidase inhibition activity ((IC50 = 36.57 μg/mL) in comparison to acarbose (IC50 = 189.57 μg/mL). Among the phenolic compounds isolated from *C. humilis* methanol extract in this study, myricetin, avicularin, pruning, quercitrin, guajavarin and isoquercitrin were accountable for their α-glucosidase activity. The *Paliurus spina-christi* mill fruit is used as an antidiabetic traditional medicine in Turkey, and a recent study showed that *n*-hexane fractions derived from the methanolic fruit extract have remarkably higher α-glucosidase inhibitory effects than acarbose with IC_50_ of 445.7 ± 8.5 and 4212.6 ± 130.0 µg/mL, respectively [85]. The phytochemical analysis of the fruit extract identified three terpenic compounds (betulin, betulinic acid and lupeol) with a higher α-glucosidase inhibitory activity than acarbose. *Gymnanthemum amygdalinum* (Delile) is another folk medicine plant that has been traditionally used in Nigeria to treat diabetes, and the flavonoid-rich fractions of its leaf extract show a substantial antidiabetic effect [86]. A recent study showed that flower methanol extract also exhibits great α-glucosidase inhibitory activity with IC_50_ greater than the positive control [87]. The flower methanolic extract fractionation with ethyl acetate solvent yield in two flavonoid compounds with luteolin showed the highest α-glucosidase activity than 2-(3,4-dihydroxy phenyl)-5,7-dihydroxy-3-methoxy-4H-chromen-4-on compared to the positive control. Polysaccharides extracted from the water extract of *Evodiae fructus*, a Chinese medicinal herb, show promising α-glucosidase inhibition activity [88]. The polar extracts of *Oryza sativa* L (black rice) bran possess potent α-glucosidase inhibitory activity [89]. The preliminary analysis of these traditional medicinal plant extracts revealed promising α-glucosidase inhibition activity, and further analysis is required to support their anti-diabetic effect.

## 4. Plant-Derived Bioactive Compounds as Potential α-Glucosidase Inhibitors

There have been reports of various plants having α-glucosidase inhibition activity. Potential AGI inhibitors have been shown to exist in a wide variety of bioactive substances that fall under several classes of secondary metabolites. Numerous secondary metabolites, including flavonoids, terpenes, phenolic acids, polysaccharides, tannins, anthocyanins, stilbene and many others, have been discovered to have α-glucosidase inhibition activity (Table 3).

### 4.1. Flavonoids

Flavonoids are polyphenolic metabolites that are often present in plants as different glycosides. Typically, they consist of two phenyl rings and one heterocyclic ring in a 15-carbon phenolic structure. They include different subgroups as flavones, isoflavones, flavans, flavanones and flavonols [141]. Flavonoids play an important role in carbohydrate metabolism. Several flavonoid molecules are found to be more effective at inhibiting α-glucosidase.

**Figure 4:**
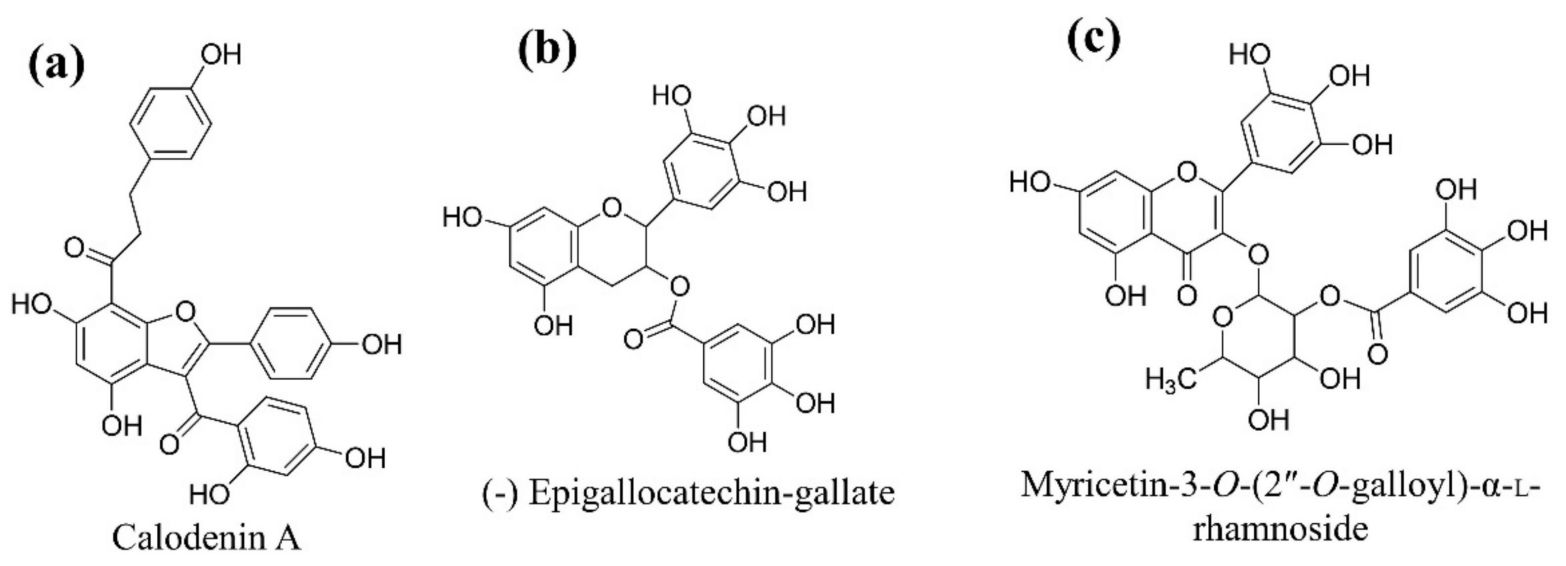
Chemical structure of some of the reported flavonoids as α-glucosidase inhibitors; (**a**) Calodenin A, (**b**) (-) Epigallocatechin-gallate, and (**c**) Myricetin-3-*O*-(2″-*O*-galloyl)-α-L-rhamnoside.

Le et al. discovered six globunones A-F, two new flavonoids and nine other known compounds that displayed potent inhibition of α-glucosidase with IC_50_ values between 0.4 and 26.6 μM. When compared to acarbose (IC_50_ = 93.6 μM), the well-known flavonoid compound Calodenin A (Figure 4a) (IC_50_ = 0.4 μM) had the greatest effect and exhibited a non-competitive mode of action during kinetic studies [114]. Similarly, Sgariglia et al. [113] isolated five polyphenolic derivatives from the bark of *Caesalpinia paraguariensis*. Among them, (-) epigallocatechin-gallate (Figure 4b) (IC_50_ = 5.2 ± 0.15 µM) showed the most significant inhibitory effect against α-glucosidase, which was almost 270-fold higher than the control acarbose (IC_50_ = 1400.0 ± 0.51 µM).

Recently, two myricetin-derived flavonols, myricetin-3-*O*-(2″-*O*-galloyl)-α-L-rhamnoside (IC_50_ = 1.32 μM) (Figure 4c) and myricetin-3-*O*-(4″-*O*-galloyl)-α-L-rhamnoside (IC_50_ = 1.77 μM), were isolated from *Morella rubra*. These compounds had a 100-fold stronger inhibitory impact on α-glucosidase enzymes than acarbose (IC_50_ = 369 μM). According to the molecular docking analysis, the flavonol–enzyme binding was improved due to pi-conjugations between the galloyl functional group and key residues of α-glucosidase at the active site, which may help to explain the significantly higher activity of these two compounds [110]. Even though the in vitro α-glucosidase assay produced encouraging results, further research must be conducted on the preclinical safety and toxicity assessment of these compounds before considering them as potential anti-diabetic medication candidates.

### 4.2. Terpenoids

Terpenoids are vitally important plant metabolites that are required for both abiotic and biotic stress resistance as well as growth and development. The structural units of terpenoids are composed of isoprene and its derivatives [142]. Based on the isoprene unit number present in the structures, they can be categorized into monoterpenoids, diterpenoids, triterpenoids and sesquiterpenoids [143]. These terpenoids possess anti-cancer, anti-inflammatory and antimicrobial properties [144]. Terpenoid-based drugs such as Taxol (anti-cancer) and Artimesinin (anti-malarial) are commercially available. Lately, researchers have been encouraged to explore terpenoid molecules for anti-diabetic properties.

**Figure 5:**
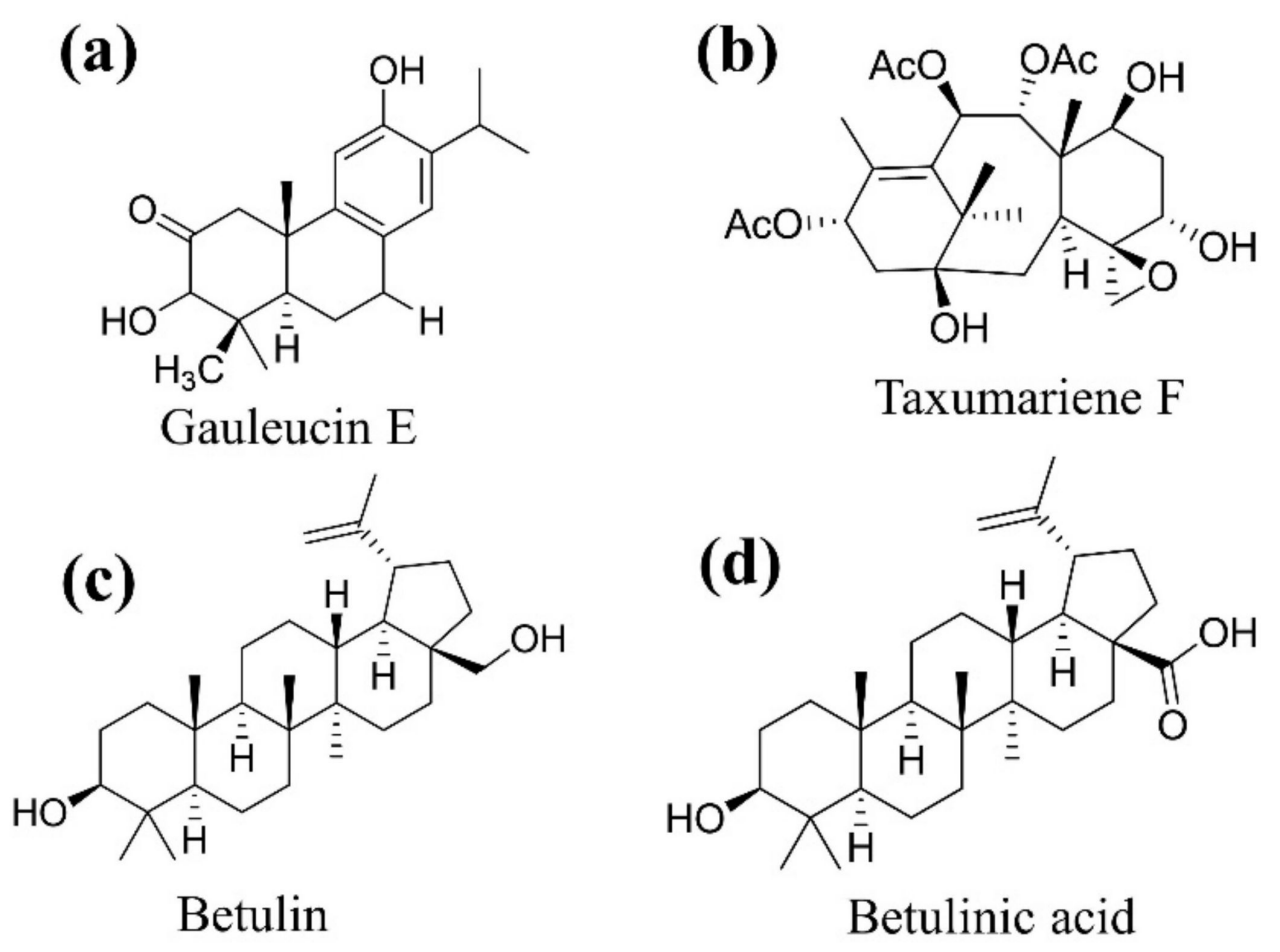
Chemical structure of some of the reported terpenoids as α-glucosidase inhibitors; (**a**) Gauleucin E, (**b**) Taxumariene F, (**c**) Betulin, and (**d**) Betulinic acid.

Two abietane-type diterpenoids, gauleucin E (Figure 5a) and margoclin derived from Gaultheria leucocarpa var. yunnanensis displayed α-glucosidase inhibitory efficacy with IC50 of 319.3 and 327.9 µM, respectively [120]. Similarly, Chen and his co-workers (Chen et al., 2020) reported seven new taxane diterpenoids, taxumarienes A–G from Taxus mairei, and assessed their α-glucosidase inhibitory activities. In comparison to the control substance acarbose (IC50 = 155.86 ± 4.12 µM), taxumariene F (Figure 5b) showed highest inhibitory effects, with an IC50 = 3.7 ± 0.75 μM. Taxumariene F’s significant inhibitory activity was ascribed to the 6/8/6 tricyclic system along with 4(20)-epoxide ring and C-9 acetoxy group. Recently, Yuca et al. evaluated the antidiabetic properties of the triterpenes isolated from Paliurus spina-christi mill fruit. Interestingly, the mixture of betulin (Figure 5c) and betulinic acid (Figure 5d) mixture (IC50 = 248 ± 12 µM) inhibited α-glucosidase 26 times better than acarbose (IC50 = 6561 ± 207 µM) [85]. In light of these findings, it may be intriguing to study the synergistic and antagonistic effects of various terpenoid compounds on α-glucosidase inhibition. Therefore, additional studies, such as kinetics studies and structure–activity relationship (SAR) studies, are essential to comprehend the underlying mechanisms for different terpenoid molecules to inhibit α-glucosidase.

### 4.3. Phenolic Acids and Their Derivatives

Phenolic acids are a group of bioactive molecules ubiquitous in plants. Their structure consists of functional carboxylic acid groups attached to aromatic phenols. Depending on the number and position of hydroxyl groups, phenolic acids can be classified into cinnamic and benzoic acid derivatives. These natural compounds are powerful antioxidants against free radicals and other reactive oxygen species (ROS) [145,146].

**Figure 6:**
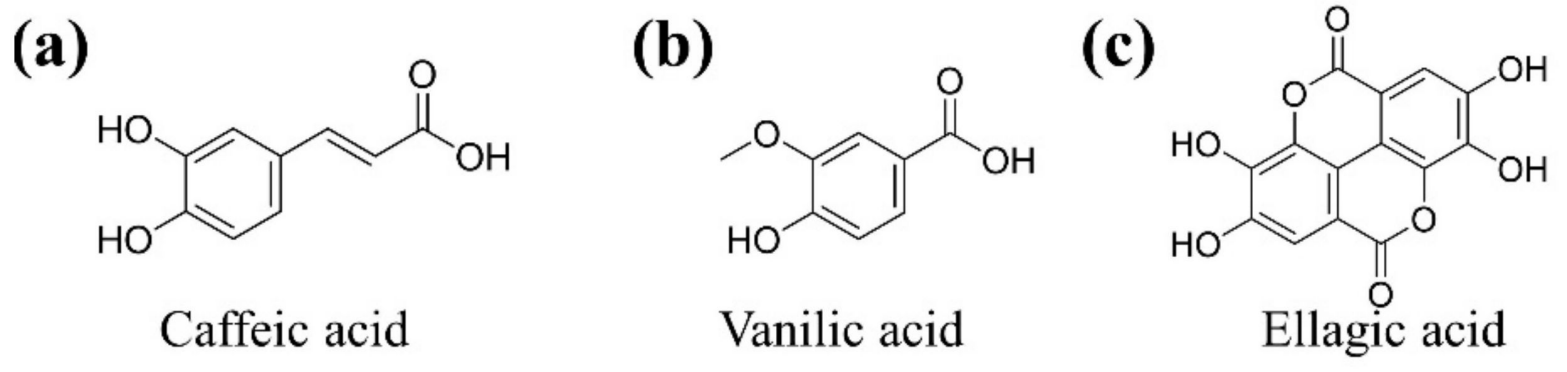
Chemical structure of some of the reported phenolic acids as α-glucosidase inhibitors; (**a**) Caffeic acid, (**b**) Vanilic acid, and (**c**) Ellagic acid.

Tergallic acid dilactone isolated from *Eugenia jambolana* exhibit potent α-glucosidase inhibitory properties with IC_50_ 5.0 ± 0.34 µM, which is 50 times higher than the positive control [121]. Aleixandre et al. [147] investigated the interactions between phenolic acids and α-glucosidase or the substrate by using different conditions such as the preincubation of phenolic acids with the enzyme or substrate and starch gelation in the presence of phenolic acid. Their studies revealed that, in comparison to phenolic acids with more hydroxyl groups, such as caffeic acid (Figure 6a) (IC_50_ = 0.39 ± 0.02 mM), phenolic acids with fewer hydroxyl groups such as vanillic acid (Figure 6b) (IC_50_ = 8.38 ± 0.01 mM) showed better inhibition against α-glucosidase. Similarly, Sgariglia et al. [113] reported ellagic acid and its derivatives isolated from *Caesalpinia paraguariensis* and performed in silico structure–activity relationship studies to evaluate the molecular interactions between α-glucosidase and the inhibitors. Ellagic acid (Figure 6c), 3-*O*-methylellagic, 3,3′-*O*-dimethylellagic acid and 3,3′-*O*-dimethylellagic-4-*O*-β-*D*-xylopyranoside show good α-glucosidase inhibition activity with IC_50_ value of 87.3, 65.1, 73.03, and 263.05 µM, respectively, which are much lower than acarbose (IC_50_ = 1400 µM). Such promising results make them a potential candidate for lead optimization. However, further research is required to assess their toxicity.

### 4.4. Polysaccharides

Polysaccharides are one of the major classes of biomacromolecules, which comprises long chains of several smaller monosaccharides. They are found in a variety of plants and animals. Growing research evidence suggests that plant-derived polysaccharides exhibit a range of biological activities with low or no toxicity [148]. Additionally, the composition of monosaccharides, glycosidic linkage and molecular weight of the polysaccharides could affect their bioactivity [149,150].

Recent evidence from the literature revealed that polysaccharides from different plant species could inhibit α-glucosidase activity [88,122,151]. A polysaccharide fraction, AXA-1, isolated from wheat bran showed a potential non-competitive mode of inhibitory effects against the α-glucosidase enzyme [123]. Zheng et al. [125] investigated the α-glucosidase inhibitory activity of several polysaccharides extracted from *Sargassum fusiforme* at different pH conditions. According to the study, SEP-7-40, which has relatively high levels of xylose and galacturonic acid and low molecular weight, exhibits a considerable inhibitory effect (IC_50_ = 0.304 mg/mL). Similarly, an acidic polysaccharide, SFP-1, isolated from *Sargassum fusiforme* inhibits α-glucosidase significantly (IC_50_ = 0.681 mg/mL) in a mixed-type inhibition mode [124]. Such potential α-glucosidase inhibitory effects shown by polysaccharides in combination with their low toxicity could be promising in the development of drugs against diabetes mellitus. Therefore, further and more organized research work is essential to understand the therapeutic role of polysaccharides in the treatment of diabetes mellitus.

### 4.5. Tannins

Tannins are polyphenolic natural compounds, which play a major role in carbohydrate metabolism [152]. They can be categorized into hydrolyzable tannins and condensed tannins. Tannins have strong anti-oxidant properties that are beneficial in the dietary and healthcare industries. Tannins are widely used in the dietary, leather and chemical industries due to their abundancy in raw materials, chemical reactivity and safe extraction [153,154].

**Figure 7:**
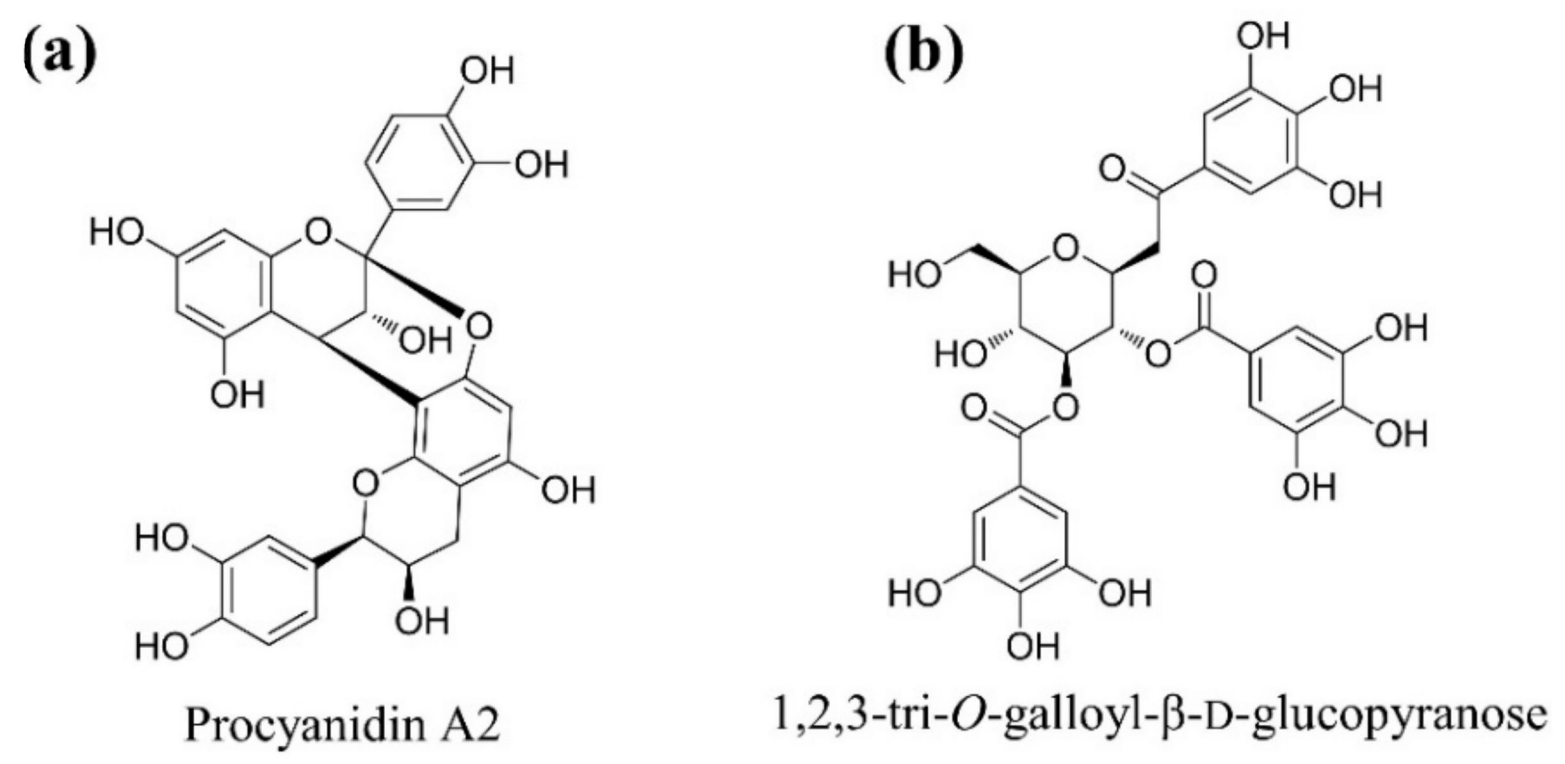
Chemical structure of some of the reported tannins as α-glucosidase inhibitors; (**a**) procyanidin A2, and (**b**) 1,2,3-tri-*O*-galloyl-β-D-glucopyranose.

Sheikh et al. [126] studied the role of tannin, procyanidin A2 (Figure 7a) in the postprandial management of diabetes mellitus. The study revealed that procyanidin A2 exhibits significant α-glucosidase inhibitory activities (IC_50_ = 0.27 ± 0.01 μg/mL). It also significantly reduced elevated G-6-Pase and mRNA levels in diabetic mice. Another study conducted by Zhang et al. [128] revealed that gallotannins isolated from *Euphorbia fischeriana* steud have antidiabetic potential. Specifically, 1,2,3-tri-*O*-galloyl-β-D-glucopyranose (Figure 7b) showed the most significant and highly selective α-glucosidase inhibitory effect. Additional SAR studies have indicated that the galloyl and glucopyranosyl groups are crucial in the inhibition of α-glucosidase. Despite these promising results, more thorough research on the mechanism and in vivo evaluations are still needed. Overcoming these drawbacks is essential in developing tannin-based significant α-glucosidase inhibitors.

### 4.6. Other Secondary Metabolites

Besides flavonoids, terpenoids, phenolic acids, tannins and polysaccharides, there are many other classes of secondary metabolites, which have been reported with significant α-glucosidase inhibitory properties. Other bioactive molecules include stilbene, anthocyanin, anthraquinone, xanthones, chalcone derivatives, pregnane glycosides, etc. [129,131,133,136,138].

**Figure 8:**
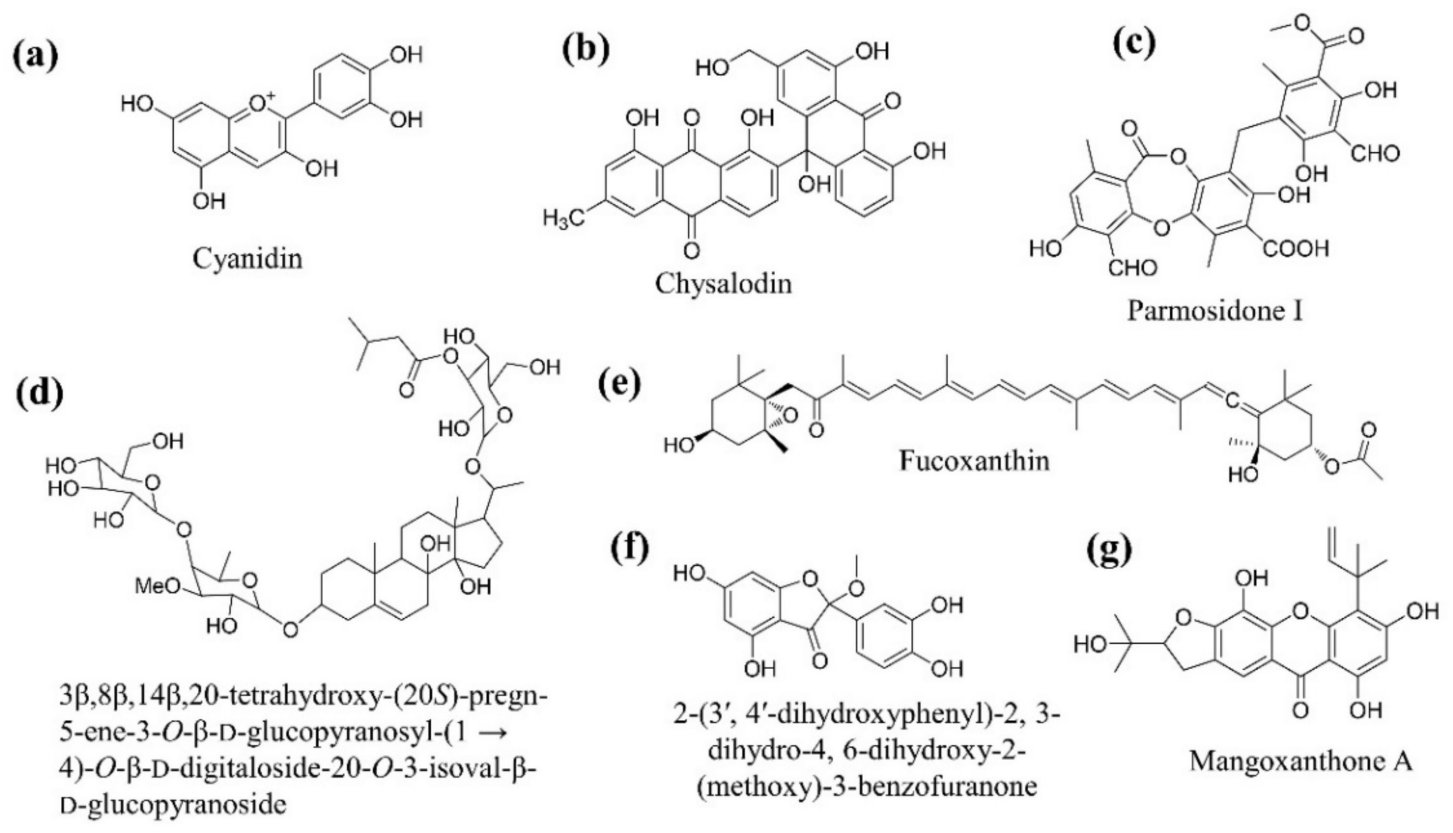
Chemical structure of some bioactive compounds reported as potential α-glucosidase inhibitors; (**a**) Cyanidin, (**b**) Chysalodin, (**c**) Parmosidone I, (**d**) 3β,8β,14β,20-tetrahydroxy-(20*S*)-pregn-5-ene-3-*O*-β-D-glucopyranosyl-(1→4)-*O*-β-D-digitaloside-20-*O*-3-isoval-β-D-glucopyranoside, (**e**) Fucoxanthin, (**f**) 2-(3′,4′-dihydroxyphenyl)-2,3-dihydro-4,6-dihydroxy-2-(methoxy)-3-benzofuranone, and (**g**) Mangoxanthone A.

J. Chen et al. [132] investigated cyanidin and its derivatives isolated from the fruit of *Cinnamomum camphora* for in vitro α-glucosidase inhibitory activities. Significantly higher inhibition was observed with cyanidin (IC_50_ = 5.293 × 10^−3^ mM) (Figure 8a) in comparison to acarbose (IC_50_ = 1.644 mM). Kim et al. [134], explored aloe vera plants and isolated various bioactive metabolites using chromatographic techniques, and they investigated their inhibitory mechanism of them on α-glucosidase. Chysalodin (Figure 8b), an anthraquinone dimer, has the greatest ability to block α-glucosidase of all of them. The kinetic analysis further showed that chysalodin competes with the substrate of α-glucosidase for binding to the active region of the receptor.

Other metabolites, depsidones isolated from lichen *Parmotrema tsavoense*, have been reported to inhibit α-glucosidase. All five new depsidones, parmosidones F–J (Figure 8c), showed significantly higher α-glucosidase inhibition with IC_50_ values ranging from 10.7 to 17.6 µM in comparison to acarbose (IC_50_ = 449 µM) [135]. Another new pregnane glycoside compound, 3β,8β,14β,20-tetrahydroxy-(20*S*)-pregn-5-ene-3-*O*-β-D-glucopyranosyl-(1→4)-*O*-β-D-digitaloside-20-*O*-3-isoval-β-D-glucopyranoside (Figure 8d), isolated from *Caralluma hexagona* Lavranos, was found to be a good α-glucosidase inhibitor (IC_50_ = 0.67 ± 0.01 µM) [137].

Another study conducted by Zaharudi and his co-workers identified fucoxanthin (Figure 8e) from *Undaria pinnatifida* as a potential α-glucosidase inhibitor, with IC_50_ of 0.047 ± 0.001 mg/mL, which is 12-fold higher than that of acarbose (IC_50_ = 0.6 ± 0.01 mg/mL) [93]. Similarly, Quan et al. [139] reported another potential α-glucosidase inhibitor from the perennial herb, *Hylotelephium erythrostictum*. The isolated bioactive compound, 2-(3′, 4′-dihydroxyphenyl)-2, 3-dihydro-4, 6-dihydroxy-2-(methoxy)-3-benzofuranone (Figure 8f) (IC_50_ = 1.8 µM) showed 457 times more inhibition than acarbose (IC_50_ = 822.9 µM) and showed a competitive mode of inhibition toward the α-glucosidase substrate. Recently, Yang et al. [140] reported new prenylated xanthone, mangoxanthone A, (Figure 8g) isolated from *Garcinia mangostana*, with moderate α-glucosidase inhibitory activity with IC_50_ of 22.74 ± 2.07 μM.

These results and conclusions, however, are derived based on the reactions to α-glucosidase in vitro and may not accurately represent the processes involved in vivo. Despite the fact that numerous bioactive substances with various structural moieties display notable α-glucosidase inhibitory activity, the pharmacodynamics behind their inhibition remain unexplored. Therefore, comprehensive and detailed research is required to assess the toxicity, potential drug interactions and long-term side effects of these reported compounds to develop them as α-glucosidase inhibitors for the treatment and management of diabetes mellitus.

## 5. Conclusions

Diabetes mellitus is a carbohydrate metabolic disorder caused by decreased insulin production or increasing insulin resistance. Herbal remedies for diabetes have been utilized in patients with insulin-dependent and insulin-independent diabetes, diabetic peripheral neuropathy, diabetic retinopathy and other diabetic-related conditions. According to the research on their potential effectiveness against diabetes, natural compounds have a significant role to play in diabetes care, which requires additional investigation for drug development and nutraceuticals from natural plant resources. However, many herbal medicines in use today have not been well researched, and some have the capacity to induce significant adverse effects and substantial drug-to-drug interactions. To understand the pharmacological activity of herbal treatments presently being used in traditional folk medicine to treat diabetes mellitus, further study is required. Although a tremendous effort has been made by scientists to analyze the antidiabetic effects of several natural products, shortcomings are still remaining. Most of the research focuses on the in vitro studies of natural products with fewer researchers conducting in vivo studies and further pharmaceutical advanced studies. Moreover, there is a need for more structural insight into the interaction between glucosidases and the promising anti-diabetic drug targets, which can have great value in new antidiabetic drug discoveries. The goal of this review paper is to summarize the most recent discoveries in research on natural products that act as α-glucosidase enzyme inhibitors. Indeed, reducing postprandial hyperglycemia is one therapeutic strategy for diabetes in its early stages. This is accomplished by slowing glucose absorption in the digestive system by inhibiting the carbohydrate-hydrolyzing enzymes α-glucosidases. Therefore, inhibitors of these enzymes reduce the rate of glucose absorption, hence dampening the postprandial plasma glucose spike. This study reviews over forty extracts collected using various solvents and more than fifty natural products. This review’s insight should contribute to the ultimate objective of discovering new therapeutic medications with greater efficacy and safety for the treatment of type 2 diabetes or to avoid hyperglycemia.

## Figures and Tables

**Table 1:** Plant extracts showing in vivo α-glucosidase inhibition activity.

Name	Extract/Part Used	Model	Type of Study	Tested Substance Dosage	Administration Route	Assessing Criterion	Effect on Animal Blood Glucose Level	Ref.
*Momordica charantia*	Methanol extract	Male albino Wistar rats	Alloxan-induced diabetes	200 mg/kg BW	Oral	Fasting blood glucose (FBG) and insulin levels	Hypoglycemic	[52]
*Artemisia absinthium* L.	Aqueous extract/leaves	Wistar rats	Alloxan-induced diabetes	200 mg/kg BW	Oral	PBGL	Hypoglycemic	[56]
Star anise	Ethyl acetate extract/ fruit	Rabbits	Alloxan-mono-hydrate-induced diabetes	250 mg/kg BW	Injection	Blood glucose levels (BGL) and body weight	Hypoglycemic	[57]
*Amomum villosum*	Water extracts/ fruit	Male SD rats	Sucrose loading test (SLT) (2 g/kg BW)	250 and 500 mg/kg BW	Oral	BGL	Hypoglycemic	[59]
*Merremia tridentata* (L.)	Ethanol extract (SE) and flavonoid-rich fraction (FF)/stem	Mice	Alloxan-induced diabetic	SE (100 mg/kg BW) and FF (50, 75 mg/kg BW)	Oral	BGL and body weight	Hypoglycemic	[60]
Lu’an guapian green tea	Methanol extract	Male mice	GTT and ITT	-	Oral	Post prandial hyperglycemia effect	Hypoglycemic	[61]
*Amomum tsao-ko*	Methanol extract	Mice	STZ-induced diabetes	100 and 200 mg/kg BW	Oral	FBG	Hypoglycemic	[62]
*Lactuca sativa*	Methanol extract	Male SD rats	STZ-induced diabetes	50, 100 and 200 mg/kg BW	Oral	BGL	Hypoglycemic	[63]
*Allium consanguineum*	Compounds 1 and 2 isolated from the plant	Albino mice	Alloxan-induced diabetic oral glucose tolerance test (OGTT)	500, 250, 125, 62.5 and 31.25 μg/kg BW	Oral	Postprandial effect	Hypoglycemic	[64]
*Amischotolype mollissima*	Ethanolic leaves extract	Swiss albino mice	OGTT (2 gm/kg BW)	250 and 500 mg/kg BW	Oral	FBG No cytotoxicity of the extract until 4000 mg/kg BW	Hypoglycemic	[65]
*Descurainia sophia*	Methanolic flower extract	Male Wistar rats	Alloxan-induced diabetic	2.25 and 4.50 g/kg BW	Oral	Blood glucose level	Hypoglycemic	[66]
Catechin and epicatechin	Phenolic extract	Male SD rats	SLT (2 g/kg BW)	20 mg/kg BW	Oral	PBG level	Hypoglycemic	[67]
*Zanthoxylum armatum*	Aqueous leaves extract	Female Swiss albino mice	Alloxan-induced diabetes	100–4000 mg/kg BW	Oral	Hypoglycemic activity	Hypoglycemic	[68]
						Lethal dose	LD_50_ 5000 mg/kg	
*Cajanus cajan (L.)*	Ethanol extract	Wistar rats	Methylglyoxal (MGO)-induced insulin resistance	10, 50 and 100 mg/kg BW	Oral	(OGTT), (ITT)/BGL	Hypoglycemic/ dose-dependent	[69]
*Rhodiola crenulata*	Ethanol extract/ root	Male SD Rat/male Kunming (KM) mice	Alloxan-induced diabetes in mice/OSTT in mice	and 400 mg/kg BW	Oral	Post carb. glucose level	Hypoglycemic	[70]
*Amomum tsao-ko* Crevost and Lemarie	Methanol extract flavonoid constituent	Male SD Rats	STZ-induced diabetes	100 mg/kg BW	Oral	Postprandial glucose level (OGTT)/FBG	Hypoglycemic	[71]
*Terfezia claveryi*	Aqueous extract Phenolic content	Male BALB/c mice	High-fat diet alloxan-induced diabetic mice	250 and 500 mg/kg BW	Oral	Blood glucose level	Hypoglycemic/ dose-dependant	[72]
*Paeonia* species	Ethanol extract (resveratrol derivatives (vateriferol or VT and trans-ε-viniferin or VF))/Seed coats	Male KM mice	Alloxan-induced diabetic mice	5, 15 and 30 mg kg BW	Oral	Oral starch tolerance test for PBG level	Hypoglycemic/ dose-dependent	[73]
*Ammodaucus leucotrichus* Coss. and Durieu	Aqueous extract/fruit	Albino Wistar rats	Alloxan diabetic rats	150 mg/kg BW	Oral	OGTT	Hypoglycemic	[74]
*Salvia polystachya* Cav.	Ethanolic extract/Terpenoid content	BALB/c mice	streptozocin–nicotinamide (STZ–NA) induced diabetes	50, 100 and 200 mg/kg BW	Oral	Oral sucrose and starch tolerance tests (OSuTT and OStTT)/OGTT and galactose tolerance test (OGaTT)/glucose load (1.5 g/kg^−1^)	Hypoglycemic/ dose-dependent	[75]
*Agathophora alopecuroides*	Methanol extract	BALB/c male albino mice	STZ-induced diabetic mice	100 and 200 mg/kg BW	Oral	RBGL and FBGL	Hypoglycemic	[76]
*Lonicera caerulea* L.	Blue honeysuckle extract	Male mice	Oral starch and maltose (2 g kg^−1^) tolerance assay	100 and 200 mg kg BW	Oral	PBG level	Hypoglycemic	[77]
*Ganoderma lucidum*	Aqueous extract of fruiting bodies (FYGL)	BKS-db (db/db) diabetic mice	OSTT (2.5 g/kg sucrose)	225, 450 and 900 mg/kg bw FYGL	Oral	PBG concentration	Hypoglycemic	[78,79]
*Colvillea racemosa*	Ethanol extract (n-butanol fraction)/leaves	Male albino rats	STZ-induced diabetes	500 mg/kg BW	Oral	FBG	Hypoglycemic	[80]
*Artemisia roxburghiana*	Aqueous ethanol extract/aerial parts	Wistar rats	STZ-NA-induced diabetes	200 and 400 mg/kg BW in a dose-dependent manner	Oral	BGL	Hypoglycemic/ dose-dependent	[81]
*Breynia distachia*	Methanol extract/aerial parts	SD rats	Alloxan-induced diabetes	150 and 300 mg/kg BW	Oral	BGL	Hypoglycemic	[82]
*Rhodomyrtus tomentosa*	Methanol extract/Leaf	Male albino Wistar rats	STZ-induced diabetes	200, 400 and 600 mg/kg BW	Oral	BGL	Hypoglycemic/ dose-dependent	[83]

**Table 2:** Summary of an in vitro α-glucosidase inhibition assay for plant extracts.

Name of Plants/Compounds	Extract/Class	Source	IC_50_	IC_50_ of Positive Control (Acarbose)	Mode or Type of Inhibition	Ref.
*Samanea saman*	Methanol extract	*Samanea saman* (leaves)	172.25 (50% inhibition)	115.2 (50% inhibition)	-	[90]
*Ganoderma hainanense*	Chloroform residue	*Ganoderma hainanense* (Fruiting body)	0.409 ± 0.041 mg/mL	-	-	[91]
*Andrographis paniculata*	Ethanolic extract	*Andrographis paniculata* (leaves)	17.2 ± 0.15 mg/mL	6.2 ± 0.33 mg/mL	-	[92]
*Undaria pinnatifida*	Acetone extract	*Undaria pinnatifida*	0.08 ± 0.002 mg/mL	0.6 ± 0.01 mg/mL	-	[93]
*Conyza canaden- sis*	Methanolic extract	*Conyza canadensis* (whole plant)	107 µg/mL	23 µg/mL	-	[94]
Cinnamon extract	Methanolic extract	*Cinnamomum zeylanicum* (Bark)	5.83 µg/mL	36.89 µg/mL	-	[95]
*Zanthoxylum armatum*	Plant extract	*Zanthoxylum armatum* (leaves)	79.82% at 0.8 mg/mL	23.83% at 0.8 mg/mL	-	[68]
*Mentha arvensis*	Methanolic extract	*Mentha arvensis* (leaves)	68% at 50 µg/µl	85% at 50 µg/µl	-	[96]
Black rice	Ethyl acetate extract	Black rice bran	47.79 ± 2.28 µg/mL	56.42 ± 4.17 µg/mL	-	[89]
	Methanolic extract		48.50 ± 0.83 µg/mL		-	
	Hexane extract		52.80 ± 1.65 µg/mL		-	
*Potentilla anserine*	Butyl alcohol fraction	*Potentilla anserine* (rhizome)	14.18 ± 0.95 µg/mL	19.15 ± 1.57 µg/mL	-	[97]
*Cyclocarya paliurus*	Plant extract	*Cyclocarya paliurus* tea (leaves)	31.5 ± 1.05 µg/mL	296.6 ± 1.06 µg/mL	-	[98]
Bound phenolic acid	Plant extract	Naked oats	0.580 ± 0.010 mg/mL	0.503 ± 0.017 mg/mL	competitive	[99]
Free phenolic acid			0.721 ± 0.014 mg/mL	0.503 ± 0.017 mg/mL	mixed	
*Nelumbo nucifera* (total flavonoids)	*Nelumbo nucifera* leaf flavonoids	*Nelumbo nucifera* (leaves)	1.86 ± 0.018 mg/mL	0.69 ± 0.047 mg/mL	-	[100]
*Evodiae fructus* (polysaccharides)	Water extract	*Evodiae fructus*	84.6% at 4 mg/mL	99.6% at 4 mg/mL	-	[88]
*Adenosma bracteosum*	Ethanolic extract	*Adenosma bracteosum* (aerial part)	26.55 µg/mL	87.94 µg/mL	-	[101]
*Lepisanthes fruticosa*	Ethanolic extract	*Lepisanthes fruticosa* (seeds)	1.873 ± 0.421 mg/mL	0.064 ± 0.002 mg/mL	-	[102]
*Symplocos cochinchinensis*	Ethanolic extract	*Symplocos cochinchinensis* (Bark)	82.07 ± 2.1 µg/mL	45 ± 1.12 µg/mL	-	[103]
*Cerasus humilis*	70% methanolic extract	*Cerasus humilis* (Sok leaf tea)	36.57 μg/mL	189.57 μg/mL	-	[84]
*Paliurus spina* *-christi Mill*	*n*-hexane sub-extract	*Paliurus spina-christi Mill.* (fruit)	445.7 ± 8.5 µg/mL	4212.6 ± 130.0 µg/mL	-	[85]
*Gymnanthemum amygdalinum*	Ethyl acetate fraction	*Gymnanthemum amygdalinum* (flower)	19.24 ± 0.12 µg/mL	73.36 ± 3.05 µg/mL	-	[87]
*Washingtonia filifera*	Methanolic extract	*Washingtonia filifera* (Seeds)	0.53 ± 0.014 µg/mL	90 ± 7.3 µg/mL	Mixed	[104]
*Crataegus pinnatifida*	Acetone extract	*Crataegus pinnatifida* (fruits)	42.35 ± 2.48 µg/mL	317.8 ± 16.36 µg/mL	-	[105]
*Chenopodium quinoa* Willd.	Ethyl acetate fraction	*Chenopodium quinoa* Willd. (Quinoa)	99.66 ± 6.0 µg/mL	336.25 ± 56.88 µg/mL	-	[106]

**Table 3:** List of in vitro α-glucosidase inhibitors reported from various plants.

Name of Plants/Compounds	Extract/ Class	Source	IC_50_	IC_50_ of Positive Control (Acarbose)	Mode or Type of Inhibition	Ref.
Catechin	Flavonoid	Commercial	1.12 ± 0.03 µM	1250 ± 35.63 µM	Competitive and reversible	[67]
Epicatechin			0.95 ± 0.02 µM	1250 ± 35.63 µM		
Naringenin	Flavonoid	Commercial	6.51 µM	49.65 µM	Competitive	[107]
Apigenin	Flavonoid	Commercial	(1.43 ± 0.02) × 10^−5^ M	(37.65 ± 0.44) × 10^−5^ M	Non-competitive	[108]
Scutellarein			(0.24 ± 0.02) × 10^−5^ M	(37.65 ± 0.44) × 10^−5^ M	Mixed	
Hispidulin			(3.21 ± 0.03) × 10^−5^ M	(37.65 ± 0.44) × 10^−5^ M		
Nepetin			(1.18 ± 0.02) × 10^−5^ M	(37.65 ± 0.44) × 10^−5^ M		
Quercetin-3-*O*-α-L-rhamnopyranoside-2″-gallate	Flavonoid	*Potentilla anserine* (rhizome)	1.05 ± 0.03 µM	28.06 ± 0.82 µM	Competitve	[97]
Quercetin-4′-*O*-glucoside	Flavonoid	*Allium cepa* (peel)	31.4 ± 0.8	51.8 ± 10.3	-	[109]
Myricetin-3-*O*-(2″-*O*-galloyl)-α-L-rhamnoside	Flavonoid	*Morella rubra* (leaves)	1.32 ± 0.17 µM	369.15 ± 6.18 µM	-	[110]
Myricetin-3-*O*-(4″-*O*-galloyl)-α-L-rhamnoside			1.77 ± 0.19 µM			
Quercetagetin-7-*O*-β-D-glucopyranoside	Flavonoid	*Rubus corchorifolius* (fruit)	4.96 ± 0.54 μM	1.93 ± 0.08 μM	Non-competitive	[111]
Vitexin	Flavonoid	Natural	52.80 ± 1.65 µM	375 ± 12.5 μM	Non-competitive	[112]
(-) epigallocatechin-gallate	Flavonoid	*Caesalpinia paraguariensis* (bark)	5.20 ± 0.15 µM	1400.00 ± 0.51 µM	Non-competitive	[113]
Calodenin A	Flavonoid	*Knema globularia* (stem)	0.4 ± 0.1 μM	93.6 ± 0.5 μM	Non-competitive	[114]
Globunone A			2.0 ± 0.1 μM			
Globunone B			1.6 ± 0.2 μM			
Globunone C			1.4 ± 0.1 μM			
Globunone F			26.6 ± 1.8 μM			
Dehydrolophirone C			3.2 ± 0.2 μM			
Lophirone P			5.6 ± 0.9 μM			
Scolopianate A	Triterpenoid	*Ganoderma hainanense*	3.4 ± 0.16 µM	489.6 ± 51.4 µM	-	[91]
Akebonoic acid	Triterpenoid	*Akebia trifoliata*	9 μM	409 μM	-	[115]
3-oxolupenal	Triterpenoid	*Nuxia oppositifolia*	62.3 ± 2.4 µg/mL	38.1 ± 3.1 µg/mL	-	[116]
Katononic acid			88.6 ± 6.2 µg/mL			
Cypaliuruside J	Triterpenoid Saponin	*Cyclocarya paliurus* (leaves)	2.22 ± 0.13 μM t	-	Non-competitive	[117]
Betulin and betulinic acid mixture	Triterpenes	*Paliurus spina-christi* Mill. (fruit)	248 ± 2 µM	6561 ± 207 µM	-	[85]
Andrographolide	Diterpenoid	Commercial	11.0 ± 0.28 mg/mL	6.2 ± 0.33 mg/mL	-	[92]
Ent-atisane-3-oxo-16β,17-acetonide	Diterpenoid	*Euphorbia antiquorum*	69.62 µM	332.5 µM	Non-competitive	[118]
Taxumariene F	Diterpenoid	*Taxus mairei*	3.7 ± 0.75 μM	155.86 ± 4.12 µM	-	[119]
Gauleucin E	Diterpenoid	*Gaultheria leucocarpa* var. *yunnanensis*	319.3 μM	387.8 μM	-	[120]
Margoclin			327.9 μM			
Tergallic acid dilactone	Polyphenols	*Eugenia jambo-lana* (seeds)	5.0 ± 0.34 µM	289.9 ± 6.67 µM	-	[121]
ellagic acid	Phenolic acid and its derivatives	*Caesalpinia paraguariensis* (bark)	87.30 ± 0.78 µM	1400.00 ± 0.51 µM	Mixed	[113]
3-*O*-methylellagic			65.10 ± 0.56 µM		Mixed	
3,3′-*O*-dimethylellagic acid			73.03 ± 0.1 µM		Non-competitive	
3,3′-*O*-dimethylellagic- 4-*O*-β-D-xylopyranoside			263.05 ± 0.12 µM		Competitive	
Vanilin	Phenolic aldehyde	Commercial	28.34 ± 0.89 mg/mL	0.52 ± 0.08 mg/mL	Mixed	[122]
AXA-1	Polysaccharides	Wheat bran	0.38 mg/mL	0.14 mg/mL	Mixed type non-competitive	[123]
WXA-1			1.17 mg/mL	0.14 mg/mL	-	
*S. fusiforme* polysaccharide (SFP-1)	Polysaccharides	*Sargassum fusiforme*	0.681 mg/mL	1.308 mg/mL	Mixed	[124]
*S. fusiforme* polysaccharide (SFP-7-40)	Polysaccharides	*Sargassum fusiforme*	0.304 mg/mL	0.657 mg/mL	Non-competitive	[125]
Procyanidin A2	Tannin	*Wendlandia glabrata*	0.47 μM	586.6 μM	-	[126]
Dieckol	Tannin	*Ecklonia cava*	0.24 ± 0.056 mM	1.05 ± 0.03 mM	-	[127]
1,2,3-tri-*O*-galloyl-β-D-glucopyranose	Gallotannins	*Euphorbia fischeriana*	15.48 ± 0.60 μM	-	Mixed	[128]
Rhaponticin	Stilbene	*Polygonum multiflorum*	0.3 μM	50.04 μM	-	[129]
Scirpusin B	Stilbene	*Cyperus rotundus* (rhizome)	94.3 ± 6.8 µM	2060 ± 97.5 µM	-	[130]
Pelargonidin-3-*O*-rutinoside	Anthocyanin	strawberries	1.69 µM	356.26 µM	Mixed	[131]
Cyanidin	Anthocyanin	*Cinnamomum camphora* (fruit)	5.291 × 10^−3^ mM	1.644 mM	Non-competitive	[132]
Alaternin	Anthraquinone	*Cassia obtusefolia*	3.45 μM	191.4 μM	-	[133]
Chysalodin	Anthraquinone	*Aloe vera*	13.4 ± 1.5 μM	124.0 ± 3.1 μM	Competitive	[134]
Parmosidone I	Depsidone	*Parmotrema tsavoense*	10.7 μM	449 μM	-	[135]
Gymnepregoside F	Pregnane glycoside	*Gymnema inodorum* (leaves)	63.7 ± 3.9% at 200 μM	-	-	[136]
3β,8β,14β,20-tetrahydroxy-(20*S*)-pregn-5-ene-3-*O*-β-D-glucopyranosyl-(1→4)-*O*-β-D-digitaloside-20-*O*-3-isoval-β-D-glucopyranoside	Pregnane glycoside	*Caralluma hexagona*	0.67 ± 0.01 mM	0.81 ± 0.86 mM	-	[137]
Mulberrofuran K	Chalcone derivatives	*Morus macroura*	1.25 μM	1428 μM	-	[138]
2-(3′,4′-dihydroxyphenyl)-2,3-dihydro-4,6-dihydroxy-2-(methoxy)-3-benzofuranone	Benzofuranone	*Hylotelephium erythrostictum*	1.8 μM	822.9 μM	-	[139]
Fucoxanthin	Xanthophyll	*Undaria pinnatifida*	0.047 ± 0.001 mg/mL	0.6 ± 0.01 mg/mL	Mixed type	[93]
Mangoxanthone A	Xanthones	*Garcinia mangostana* (pericarp)	29.06 ± 1.86 μM	-	-	[140]

## Data Availability

Not applicable.
